# An analysis of spontaneous adverse drug reaction reports of psychiatric patients stratified by reporter type provides differentiated data

**DOI:** 10.1192/j.eurpsy.2025.553

**Published:** 2025-08-26

**Authors:** D. Dubrall, P. Christ, M. Schmid, B. Sachs, C. Scholl

**Affiliations:** 1 University Hospital Bonn; 2 Federal Institute for Drugs and Medical Devices, Bonn; 3 University Hospital RWTH Aachen, Aachen, Germany

## Abstract

**Introduction:**

In Germany, psychiatric drugs are one of the most frequently prescribed drug class with more than two billion defined daily doses. As a consequence, a high number of patients carries a potential risk to develop an adverse drug reaction (ADR) to these drugs. It is known that spontaneous ADR reports from physicians, pharmacists and consumers differ regarding the drugs reported as suspected and the ADRs most frequently described. Most of the studies investigating differences in ADR reports from different reporter types are based on datasets without stratification into specific drug classes.

**Objectives:**

The aim of our study was to analyse whether the reported psychiatric drugs, ADRs and types of psychiatric patients with regard to their psychiatric history and diagnosis differed between spontaneous ADR reports related to psychiatric drugs submitted by physicians, pharmacists and consumers.

**Methods:**

Overall, we analysed 2,606 spontaneous ADR reports from physicians, 1,222 from pharmacists and 4,218 from consumers from Germany received for adults in which antidepressants, antipsychotics and mood stabilizers were reported as suspected/interacting. Comparative analyses between the ADR reports of the three reporter types concerning the psychiatric drugs reported as suspected/interacting, the histories of the patients and the reported ADRs were performed. Odds ratios (OR) with their 95% confidence intervals (CI) were calculated by a two-by-two table design.

**Results:**

Consumers more commonly reported ADRs related to the drug classes of other antidepressants compared to pharmacists and physicians. In contrast, physicians reported ADRs occurring after the use of other antipsychotics more frequently compared to the respective other reporter types. Concerning the ADRs, sexual dysfunctions were more commonly described in ADR reports from consumers, laboratory investigations and their results more often in ADR reports from physicians and product quality issues in ADR reports from pharmacists. With regard to the psychiatric histories of the patients, ADR reports from consumers more often contained patients with anxiety disorders and ADR reports from physicians patients with schizophrenia and bipolar disorders.

**Conclusions:**

Our study underlines the value of considering the ADR reports from the three different reporter types as these reports complement each other. This approach provides a more complete analysis of the drug safety and may therefore allow risk minimization measures, which integrate and specifically consider the perspectives of the respective reporter types if necessary.

**Disclosure of Interest:**

None Declared

